# An Analysis of the Clinical and Radiological Outcomes of Fragment-Specific Fixation of Intra-articular Distal End Radius Fractures

**DOI:** 10.7759/cureus.71597

**Published:** 2024-10-16

**Authors:** Pratik T Gundecha, Ishan Shevate, Rahul Salunkhe

**Affiliations:** 1 Orthopedics, Dr. D. Y. Patil Medical College, Hospital and Research Centre, Dr. D. Y. Patil Vidyapeeth (Deemed to be University), Pune, IND

**Keywords:** distal radius fractures, fixation, fragment-specific fixation, functional recovery, periarticular fragments

## Abstract

Background and objective

Distal radius fractures (DRFs) present unique challenges for fixation, as they often involve small, periarticular fragments that require advanced techniques to achieve proper anatomical alignment and functional recovery. This study aimed to investigate the efficacy of fragment-specific fixation in managing these fractures.

Methods

We conducted a longitudinal study involving 57 patients with fresh intra-articular DRFs. The participants, aged 18-80 years, were evaluated through comprehensive radiological assessments, including X-rays and CT scans, alongside clinical examinations. Ethical approval and informed consent were obtained for all procedures.

Results

The sample included 57 patients; 57.89% were male and 42.11% were female. The most common occupations were housewives (33.33%) and business professionals (19.30%). Injuries were nearly equally distributed between the left (47.37%) and right (52.63%) sides, with road traffic accidents accounting for 52.63% of cases. Radiological evaluations indicated significant improvements in volar tilt, from 10.38 ± 1.46 degrees postoperatively to 9.57 ± 1.67 degrees after three months, and in radial inclination, from 21.09 ± 1.72 degrees to 20.28 ± 2.04 degrees. Clinical outcomes, as measured by Green and O'Brien and Gartland and Werley scores, showed a substantial recovery, with 75.44% of patients achieving an excellent rating with no poor outcomes.

Conclusions

Based on our findings, fragment-specific fixation for DRFs is highly effective, leading to significant improvements in both radiological and functional outcomes. The technique helped maintain anatomical alignment and facilitated bone healing and we recommend its adoption as a standard treatment for complex DRFs. We also recommend future studies investigating its long-term outcomes and comparing this approach with other fixation methods to optimize patient care further.

## Introduction

Intra-articular fractures of the distal radius are some of the most common injuries encountered in orthopedic practice, and they can have a substantial impact on wrist and hand function [1]. These fractures, which involve the articular surface of the distal radius, are particularly challenging due to the complexity of the joint anatomy and the necessity to restore both the anatomical alignment and joint congruence to achieve optimal functional outcomes [2].

These fractures account for approximately 17% of all fractures treated in emergency departments globally and are a significant concern in both developed and developing nations [3]. Several factors contribute to the increasing incidence of distal radius fractures (DRFs), including lifestyle, environmental influences, rising life expectancy, increased childhood obesity, and higher rates of osteoporosis in the elderly population [4]. Research indicates that DRFs are most common in pediatric males and postmenopausal women, with a significant occurrence also noted in young adult men aged 19-49 years [5]. In younger patients, DRFs typically result from high-energy trauma, whereas in the elderly, these fractures are more often due to low-energy trauma [6].

The treatment of these complex fractures has progressed from primarily non-invasive alignment and immobilization of the bone to advanced techniques like open reduction, which involves intraoperative alignment of the bone, and internal fixation with plates. plating [7]. These developments in treating complex fractures now encompass a range of options [8]. The choice of treatment is influenced by several factors, including the type of fracture, the degree of comminution, patients' age, and their socioeconomic status [9]. However, despite these advances, a reliable and consistent method for restoring joint congruity, anatomical alignment, and achieving optimal functional outcomes is still not well established.

This study aimed to assess the clinical and radiological outcomes of using fragment-specific fixation for treating intra-articular fractures of the distal end of the radius. The study provides a detailed analysis of patient outcomes, focusing on the restoration of anatomical alignment, joint congruence, and functional recovery.

## Materials and methods

Study design and setting

This longitudinal study was conducted at the Dr. D.Y. Patil Medical College, Pune, and spanned the period from February 2022 to August 2024. The study focused on patients presenting with fresh intra-articular DRFs. Ethical approval was obtained from the Institutional Ethics Committee (IESC/PGS/2022/97), and informed consent was secured from all participants before their inclusion in the study.

Study population

A total of 57 patients participated in the study. Patients aged between 18 and 80 years presenting with fresh distal end radius fractures, and those with intra-articular fractures of the distal end radius were included. Patients with compound fractures of the distal end radius, those who were medically unfit, individuals with malunited distal end radius fractures, and patients aged below 18 years or above 80 years were excluded.

Procedure

Patients diagnosed with distal end radius fractures underwent detailed radiological assessments, including X-rays and CT scans, to aid in preoperative planning. Depending on the type of fracture, the fracture fragments were exposed using the appropriate surgical approach. Fracture reduction and fixation were performed under direct visualization and verified intraoperatively using C-arm fluoroscopy. Closure was executed following fragment-specific fixation. Postoperative follow-up visits were scheduled immediately, and subsequently at three weeks, and six weeks, and continued until the radiological union of the fractures was confirmed. Functional outcomes were assessed using Green and O'Brien (Cooney’s modification) and Gartland and Werley scores [10].

Data collection and analysis

Data entry and analysis were conducted using Microsoft Excel and WinPepi software. Categorical variables were presented as frequencies and percentages, while continuous variables were expressed as means and standard deviations (SD). Statistical analyses were performed using the Chi-square test, Student's t-test, ANOVA, and Pearson correlation. A p-value of less than 0.05 was considered statistically significant, with a 95% confidence interval applied to all statistical tests.

## Results

The study included 57 patients: 33 (57.89%) males and 24 (42.11%) females. The most common occupations were housewives (19 patients, 33.33%) and business professionals (11 patients, 19.30%). Injuries were nearly equally distributed between the left side (27 patients, 47.37%) and the right side (30 patients, 52.63%), with road traffic accidents accounting for 30 cases (52.63%). Types C2 and C3 were the most common, comprising 16 cases (28.07%) each (Table 1).

**Table 1:** Patient demographics and injury characteristics

Parameter	Category	Cases	Percentage
Sex	Female	24	42.11%
	Male	33	57.89%
Occupation	Business	11	19.30%
	Driver	4	7.02%
	Housewife	19	33.33%
	Mechanic	3	5.26%
	Retired	1	1.75%
	Shopkeeper	3	5.26%
	Student	8	14.04%
	Sweeper	1	1.75%
	Tailor	2	3.51%
	Teacher	1	1.75%
	Worker	4	7.02%
Side of injury	Left	27	47.37%
	Right	30	52.63%
Mode of injury	Fall	27	47.37%
	Road traffic accident	30	52.63%
AO classification	B1	2	3.51%
	B2	7	12.28%
	B3	6	10.53%
	C1	10	17.54%
	C2	16	28.07%
	C3	16	28.07%

Radiological evaluations demonstrated notable improvements in volar tilt, which changed from 10.38 ± 1.46 degrees post-operatively to 9.57 ± 1.67 degrees after three months. Radial inclination also showed improvement, from 21.09 ± 1.72 degrees to 20.28 ± 2.04 degrees over the same period. Clinical outcomes, assessed using the Green and O'Brien score, revealed substantial recovery with 75.44% of patients achieving an excellent rating, and no patients exhibiting poor outcomes (Table 2).

**Table 2:** Clinical and radiological outcomes The Student's t-test was used to calculate the p-value; p<0.05 is statistically significant SD: standard deviation

Outcome	Postoperative mean ± SD	3-month mean ± SD	P-value
Volar tilt (degrees)	10.38 ± 1.46	9.57 ± 1.67	0.006
Radial inclination (degrees)	21.09 ± 1.72	20.28 ± 2.04	0.023
Green O'Brien score, n (%)			-
Excellent	-	43 (75.44%)	
Good	-	13 (22.81%)	
Fair	-	1 (1.75%)	
Poor	-	0 (0.00%)	

## Discussion

The management of intra-articular DRFs has evolved significantly over the years, focusing on improving both functional and radiological outcomes [11]. Our study demonstrates that careful and well-planned surgical intervention, particularly when utilizing fixation techniques, can lead to substantial improvements in both volar tilt and radial inclination, as well as favorable clinical outcomes as measured by the Green and O'Brien score.

**Figure 1:**
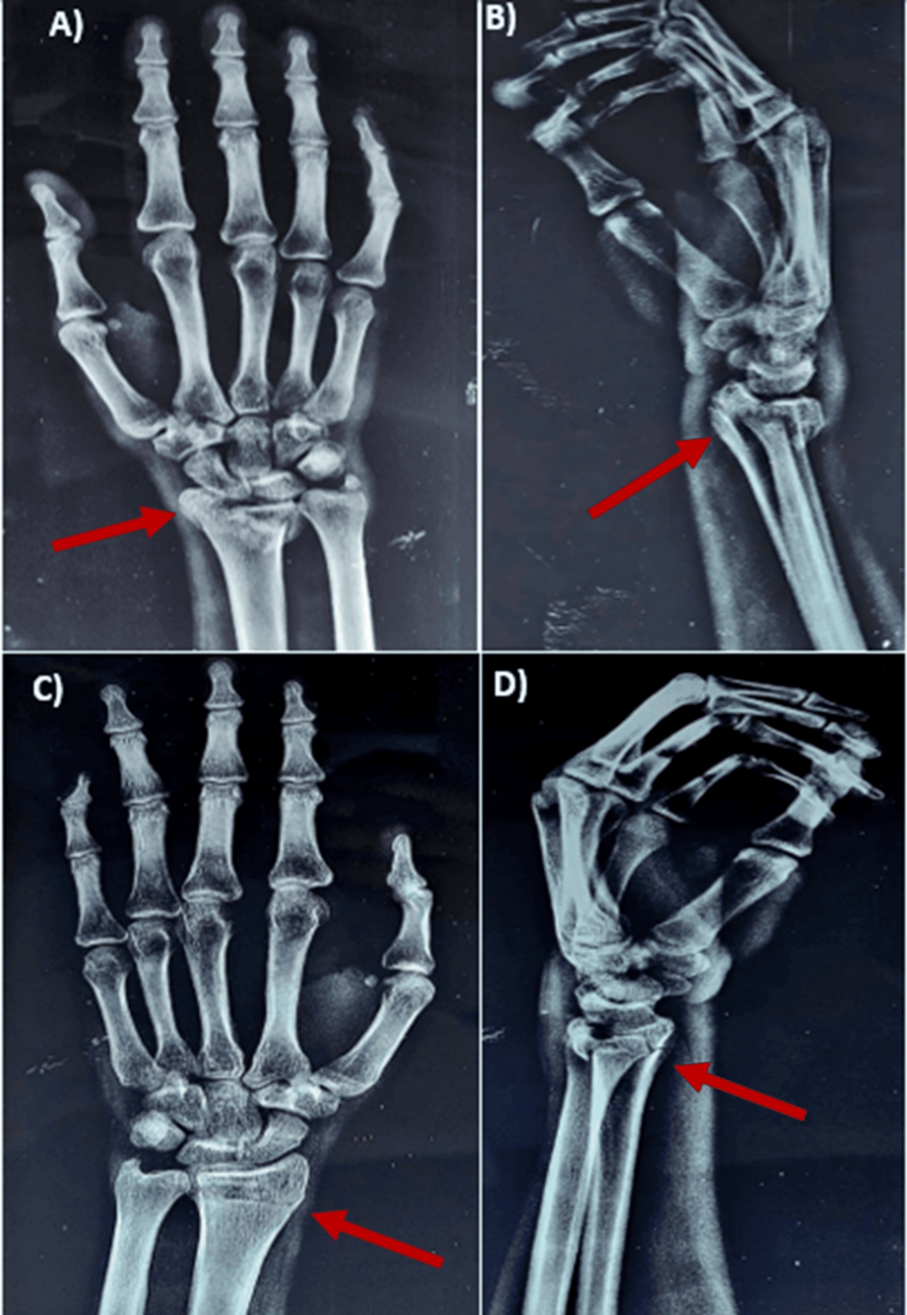
Preoperative X-rays A) Preoperative X-ray of the right wrist in AP view showing distal end radius fracture. B) Preoperative X-ray of the right wrist in lateral view showing distal end radius fracture. C) Preoperative X-ray of the left wrist in AP view showing distal end radius fracture. D) Preoperative X-ray of the left wrist in lateral view showing distal end radius fracture

The present study demonstrated that fragment-specific fixation significantly improves clinical and radiological outcomes in distal radius fractures. The high proportion of patients achieving an excellent Green and O'Brien score aligns with previous studies, indicating the efficacy of this technique in restoring functional outcomes [12-14]. The complexity of the fractures (Figure 1) and management strategy in our study concur with the findings reported by Tang et al., who highlighted the challenges of managing complex DRFs and the importance of precise anatomical restoration for optimal recovery [15].

**Figure 2:**
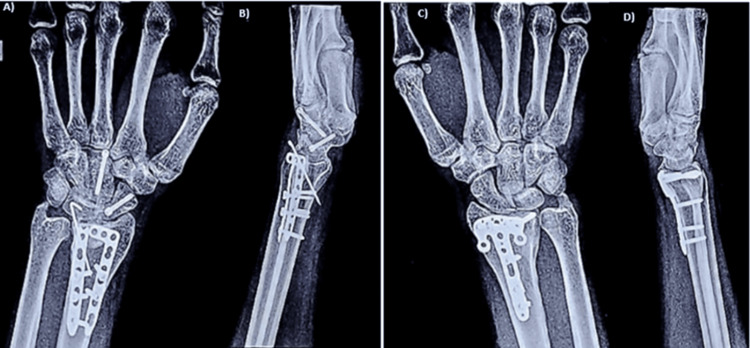
Postoperative X-rays A) Postoperative X-ray of the right wrist in AP view. B) Postoperative X-ray of the right wrist in lateral view. C) Postoperative X-ray of the left wrist in AP view. D) Postoperative X-ray of the left wrist in lateral view

Present radiological outcomes showed improvements in volar tilt and radial inclination (Figure 2), consistent the with findings by Chhabra and Yildirim (2021), who reported that maintaining anatomical alignment is crucial for the successful management of these fractures [16]. The use of fragment-specific fixation ensured proper alignment and facilitated bone healing, supporting the findings by Bowers et al., who also noted improved outcomes with this approach compared to traditional methods [17]. Another study by Orbay and Fernandez showed that volar locking plates offer superior stability and alignment, leading to better functional recovery [18].

**Figure 3:**
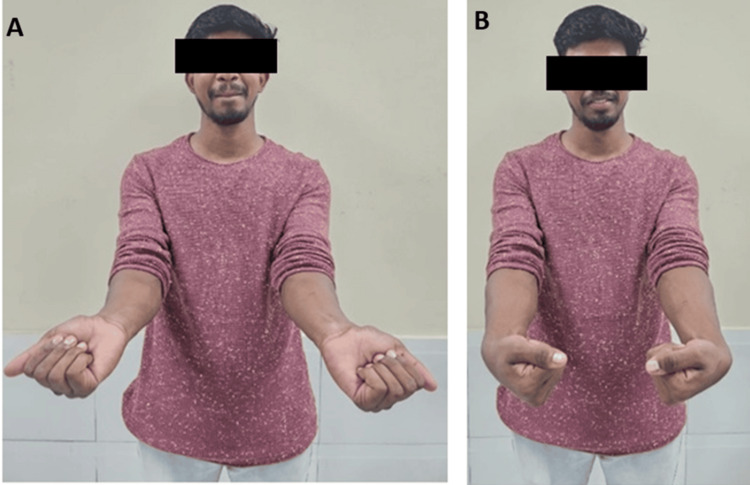
Postoperative clinical outcome A) Ulnar deviation. B) Radial deviation of the bilateral wrist joint

We observed a significant improvement in anatomical alignment and bone healing achieved through fragment-specific fixation (Figure 3). The X-ray in radial deviation, which can show the alignment and recovery progress of the wrist in a specific position, helped us assess the restoration of radial inclination and joint congruence. The X-ray of ulnar deviation provided a view of the wrist's healing in another position, aiding in the evaluation of volar tilt and overall anatomical alignment.

The present study also highlights the demographic trends and occupational risks associated with DRFs. The presence of a substantial number of housewives and business professionals among the patients suggests that these fractures are not limited to high-risk occupations but also affect those engaged in everyday activities. This finding is consistent with the study by Kapoor et al., which indicated a high incidence of DRFs in both active and non-active populations [11].

Limitations

Despite the positive outcomes, our study has limitations that should be considered. The relatively small sample size and single-center design may limit the generalizability of our findings. Furthermore, the follow-up period of three months may not fully capture the long-term outcomes, such as the potential development of post-traumatic arthritis, which is a known complication of intra-articular fractures [19]. Future research should involve larger, multicenter studies with extended follow-up to validate these findings and further explore the long-term effects of various treatment modalities.

## Conclusions

Fragment-specific fixation for DRFs proved to be highly effective, demonstrating significant improvements in both radiological and functional outcomes. The technique helped maintain anatomical alignment and facilitated bone healing, supporting its adoption as a standard treatment for complex DRFs. Overall, our findings provide valuable insights into the management of these complex fractures and suggest that advanced surgical techniques can significantly enhance patient recovery.
